# Blunted Sodium Excretion in Response to a Saline Load in 5 Year Old Female Sheep Following Fetal Uninephrectomy

**DOI:** 10.1371/journal.pone.0047528

**Published:** 2012-10-15

**Authors:** Yugeesh R. Lankadeva, Reetu R. Singh, Lucinda M. Hilliard, Karen M. Moritz, Kate M. Denton

**Affiliations:** 1 Department of Physiology, Monash University, Victoria, Australia; 2 Department of Anatomy and Developmental Biology, Monash University, Victoria, Australia; 3 School of Biomedical Sciences, University of Queensland, St Lucia, Australia; University of Louisville, United States of America

## Abstract

Previously, we have shown that fetal uninephrectomy (uni-x) causes hypertension in female sheep by 2 years of age. Whilst the hypertension was not exacerbated by 5 years of age, these uni-x sheep had greater reductions in renal blood flow (RBF). To further explore these early indications of a decline in renal function, we investigated the renal response to a saline load (25 ml/kg/40 min) in 5-year old female uni-x and sham sheep. Basal mean arterial pressure was ∼15 mmHg greater (P_Group_<0.001), and sodium excretion (∼50%), glomerular filtration rate (∼30%, GFR) and RBF (∼40%) were all significantly lower (P_Group_<0.01) in uni-x compared to sham animals. In response to saline loading, sodium excretion increased significantly in both groups (P_Time_<0.001), however this response was blunted in uni-x sheep (P_GroupxTime_<0.01). This was accompanied with an attenuated increase in GFR and fractional sodium excretion (both P_GroupxTime_<0.05), and reduced activation of the renin-angiotensin system (both P<0.05), as compared to the sham group. The reduction in sodium excretion was associated with up-regulations in the renal gene expression of NHE3 and Na^+^/K^+^ ATPase α and β subunits in the kidney cortex of the uni-x compared to the sham animals (P<0.05). Notably, neither group completely excreted the saline load within the recovery period, but the uni-x retained a higher percentage of the total volume (uni-x: 48±7%; sham: 22±9%, P<0.05). In conclusion, a reduced ability to efficiently regulate extracellular fluid homeostasis is evident in female sheep at 5 years of age, which was exacerbated in animals born with a congenital nephron deficit. Whilst there was no overt exacerbation of hypertension and renal insufficiency with age in the uni-x sheep, these animals may be more vulnerable to secondary renal insults.

## Introduction

A congenital nephron deficit has been associated with the development of renal insufficiency, hypertension and increased risk of developing cardiovascular disease [1], [2]. However a critisism that can be directed at the majority of these studies is that they have focused on early adulthood and not examined this relationship in more clinically relevant aged cohorts. Moreover, the few studies that have examined the impact of fetal programming in older cohorts have not on the whole revealed evidence of disease progression with age [3]–[5]. Thus, the question of whether early indications of increased arterial pressure and lower renal function in models of nephron deficit track and progress to cardiovascular or renal disease with age remains to be determined.

Although many developmental programming models illustrate an association between low nephron endowment and hypertension, determining the direct consequence of a nephron deficit on the development of hypertension is difficult. This is due to most *in utero* perturbations commonly employed in fetal programming models also affecting the development of multiple organs [6]–[8]. To determine the direct consequences of a nephron deficit on arterial pressure and renal function, we have established an ovine model of fetal uninephrectomy (uni-x). Nephrectomy is performed during a period of nephrogenesis at 100 days of the 150 day gestational period in sheep, which corresponds to the second trimester during human gestation. The ovine fetus has a similar pattern of permanent (metanephric) kidney development when compared to that of the human fetus and both species complete nephrogenesis by approximately 90% of their gestational period [9]. Thus, the sheep is a suitable model to study the long-term cardiovascular repercussions of being born with a low nephron endowment. Using this model, we have previously demonstrated that male uni-x sheep have a ∼30% reduction in nephron number [10], low renin hypertension from as early as 6 months of age [11], altered responses to angiotensin II [12] and a reduced ability to excrete a saline load [13]. In combination, these findings suggest that regulation of renal function in young sheep with a congenital nephron deficit is disturbed and that the hypertension is directly associated with the reduction in nephron endowment, at least in younger males.

There is significant evidence revealing that sex-differences exist in the fetal programming of hypertension [1], [14], [15]. Post-puberty, females are protected from programmed increases in arterial pressure as compared to males [16], [17]. This effect is lost with age and is likely to be associated with a decline in estrogen/androgen ratio [18]. Indeed, we have previously demonstrated that female uni-x sheep with intact ovaries do not develop hypertension until 2 years of age [19], unlike males [11] or ovariectomised females [20], which have elevated arterial pressure at 6 months of age. These findings strongly support a role for sex hormones in the sexual dimorphism apparent in this model of fetal programming of adult disease. During normal ageing there is a progressive loss of nephrons and this is associated with an age-dependent fall in renal function [21]–[24]. This decline in renal function occurs earlier in males than females and has been linked to loss of nitric oxide production [25]. Similarly sex-differences in the fetal programming of renal disease have been reported [26]. Recently, we have shown that the hypertension and reduction in glomerular filtration rate (GFR) do not appear to be exacerbated at 5 years of age in intact uni-x female sheep [19]. This being said, these uni-x female sheep demonstrated a greater reduction in the renal blood flow (RBF), as well as early signs of cardiovascular disease as evidenced by left ventricular hypertrophy and reductions in cardiac function by 5 years of age [19]. Hence to explore these early indications of a decline in renal function in more detail, we hypothesised that in female uni-x sheep at 5 years of age, the capacity to excrete a saline load would be reduced and that this would, in part, be related to a lack of responsiveness of the renin-angiotensin system (RAS). Thus, the aim of the current study was to examine arterial pressure and renal function responses to an isotonic saline load (2.5% body weight) in 5-year old female uni-x and sham sheep.

## Materials and Methods

### Animals

All experiments were approved by the Monash University, School of Biomedical Sciences Animal Ethics Committee, and were carried out in agreement with the guidelines of the National Health and Medical Research Council of Australia. Merino ewes carrying female fetuses of known gestational age underwent surgery at 100 days post-conception, as described previously [20], [27]. The left renal artery, vein and ureter were ligated and the kidney was cleared from the surrounding fat (uni-x group, n = 7). In seven fetuses, the kidney was cleared from the surrounding fat but left intact (sham group, n = 7). Following surgery, ewes were housed in large pens with free access to food and water for 2 weeks before being returned to the farm. After spontaneous birth, lambs were kept with their mothers until weaned at 4 months of age. At 5 months of age, all lambs underwent surgery, where the right carotid artery was exteriorized into a skin fold to form a carotid arterial loop [28], and following recovery the lambs were returned to pasture. As previously reported, at 1 and 2 years of age basal blood pressure and renal function were measured in these sheep [19]. At 5 years of age, female uni-x and sham animals were brought into the laboratory and placed in individual metabolic cages. Animals were fed a diet of lucerne chaff (1 kg) and allowed access to 5 litres of water at 17.00 h daily. Experiments commenced following a week of acclimatization.

### Measurements of Blood Pressure and Renal Function

For direct measurements of arterial pressure, a tygon catheter was inserted into the carotid arterial loop under local anaesthesia. The tygon catheter was then connected to a pressure transducer (TD XIII; Cobe) for measurement of mean arterial pressure (MAP) and heart rate (HR). The left and right jugular veins were catheterized for infusion of drugs and to enable delivery of the saline load. All animals were acutely instrumented with a Foley bladder catheter (size 12, 30cc, Euromedical, Malaysia) in order to allow continuous collection of urine. The bladder catheters were flushed with antibiotics (Neomycin (Jurox, 4.5 ml (200 mg/ml)) daily to minimize the risk of bladder infections. On the day of each renal experiment, animals received a bolus and continuous intravenous infusion of ^51^Chromium-ethylenediamine-tetra-acetic acid (^51^Cr EDTA, 15 µCi bolus +15 µCi/h, i.v. Amersham International) and para-aminohippuric acid (PAH, 4.8 mg/kg bolus +750 mg/h,i.v. Sigma Aldrich). An hour equilibration period was allowed for these markers to reach steady state in the plasma. GFR was determined via the clearance of ^51^Cr EDTA and effective renal plasma flow (ERPF) and renal blood flow (RBF; ERPF/(1-hematocrit)) were determined via clearance of PAH. Filtration fraction (FF) was determined as GFR/ERPF. Filtered sodium load was calculated as (plasma [Na^+^]×GFR). Urinary sodium excretion (U_Na+_V) was calculated as (Urinary [Na^+^]×Urine flow (UF)) and fractional sodium excretion (%) (FE_Na+_ was calculated as ((U_Na+_V/filtered load of sodium)×100).

### 6-hour Time Control Experiment

On the day following the insertion of the catheters, a 6 hour time control experiment was performed to examine basal cardiovascular and renal function over time. Following an hour equilibration period, MAP and HR were acquired every 10 s over the next 6 hours, the data is reported as hourly averages. Simultaneously, urine was collected every 30 minutes and heparinised blood samples (5 ml) collected at every mid-point of each urine collection in order to determine renal function.

### Response to an Acute Saline Load

Two days following the time control experiment, the response to a 2.5% body weight saline load was examined. MAP and HR were recorded continuously throughout the course of the experiment. First, basal urine samples were collected at 30 minute intervals, with arterial blood samples collected at the mid-point of urine collections for 2 hours. Following these control measurements, animals were infused intravenously with 25 ml/kg body weight of 0.9% isotonic saline over a 40 minute period. During the saline load, blood and urine samples were collected at 10 minute intervals. Following the saline load, continuous measurements of cardiovascular and renal function were made at 30 minute intervals over a 3 hour recovery period. Additional arterial samples (5 ml) were collected into a chilled tube containing EDTA to assess plasma renin activity (PRA). PRA samples were collected at 60 and 120 minutes during the control period, at the end of the 40 minutes of saline loading and at the end of the 3 hour recovery period.

### Blood and Urine Samples

Plasma and urine PAH concentrations were assessed using a rapid microplate assay [29] and ^51^Cr EDTA levels were determined using a gamma counter (PerkinElmer Wizard 1470). Urinary sodium concentrations were measured using a RapidChem 744 Electrolyte analyser; and PRA was measured by radio-immunoassay (Prosearch International, Melbourne, Australia).

### Gene Expression

Three weeks following the completion of all experiments, animals were humanely euthanized (pentobarbitone, Lethabarb®). A 0.5 cm slice taken from one half of the right kidney, in transverse plane, and subdivided into cortex and medulla (inner and outer combined). These samples were then homogenised and RNA extracted for determining gene expression of the Na^+^/K^+^ ATPase transporter (α, β and γ subunits), NHE3 (apical sodium hydrogen exchanger type 3) and ENaCs (epithelial Na^+^ channels; α and β subunits) by real-time PCR using a comparative cycle of *C_T_* (threshold fluorescence) method as previously described [13].

### Statistical Analysis

All data are reported as mean ± SEM. All renal variables were corrected for body weight (bw). Kidney and body weights in this cohort of animals have been previously reported [19]. A two-tailed Student’s t test was used to compare basal differences between the sham and uni-x groups. Analysis of variables during the 6-hour time control experiment and in response to the saline load were assessed using repeated measures ANOVA with factors group (P_Group;_ sham or uni-x), time (P_Time_) and their interaction (P_GroupxTime_), where appropriate a t-test with a Bonferroni correction to conservatively adjust for multiple comparisons was performed. Statistical analysis was performed using GraphPAD PRISM 5.03 for Windows.

## Results

### 6-hour Time Control Experiment

**Figure 1:**
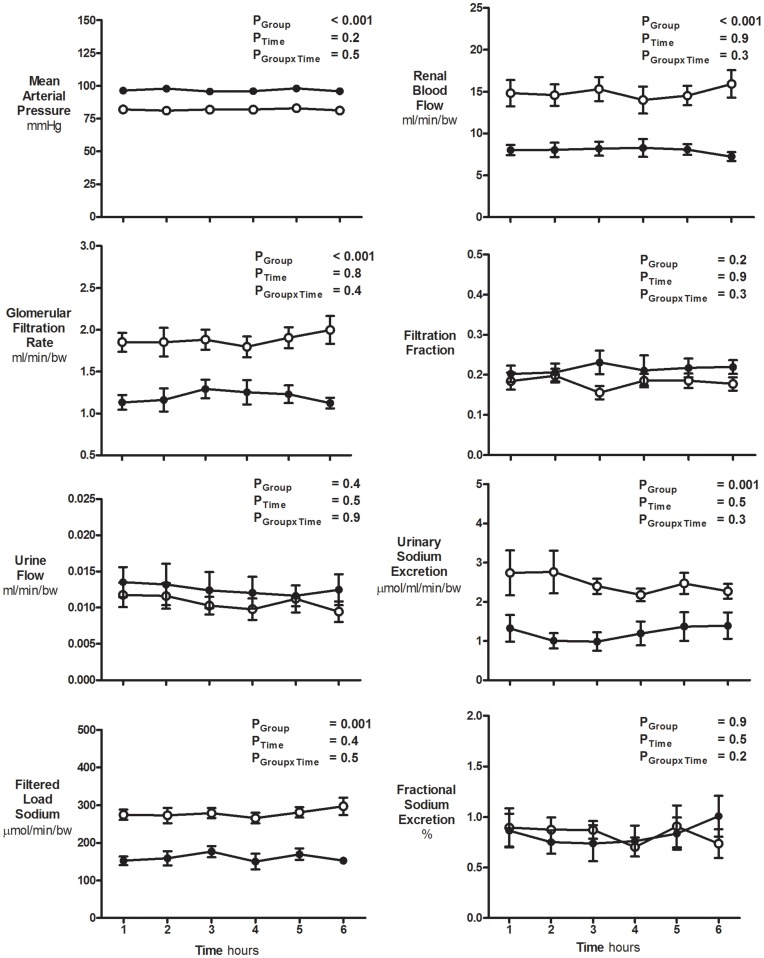
Basal cardiovascular and renal function during the time control study. Variables (mean ± S.E.M) were measured at hourly intervals over a 6 hour period in 5 year old sham (n = 7; open circles) and uni-x (n = 7; closed circles) female sheep. All renal variables are presented as absolute values corrected for body weight. P values represent the results of a repeated measures ANOVA, with factors group, (sham, uni-x), time and their interaction.

Body weights were not significantly different between sham (61±2 kg) and uni-x (58±2 kg) female sheep at 5 years of age. Basal cardiovascular and renal variables did not change significantly throughout the 6-hour time control experiment in either the sham or uni-x female sheep (all P_Time_>0.05; Figure 1). Basal MAP averaged 97±1 mmHg across the 6 hour period in the uni-x sheep as compared to 82±2 mmHg in the sham sheep (P_Group_<0.001; Figure 1). Basal FF, UF and FE_Na+_% were similar between the two experimental groups (Figure 1). However in the uni-x-sheep basal U_Na+_V (∼50%), GFR (∼30%) and RBF (∼30%) were all significantly lower as compared to the sham group (all P_Group_<0.01; Figure 1).

### Response to Acute Saline Load

#### Mean arterial pressure and heart rate

**Figure 2:**
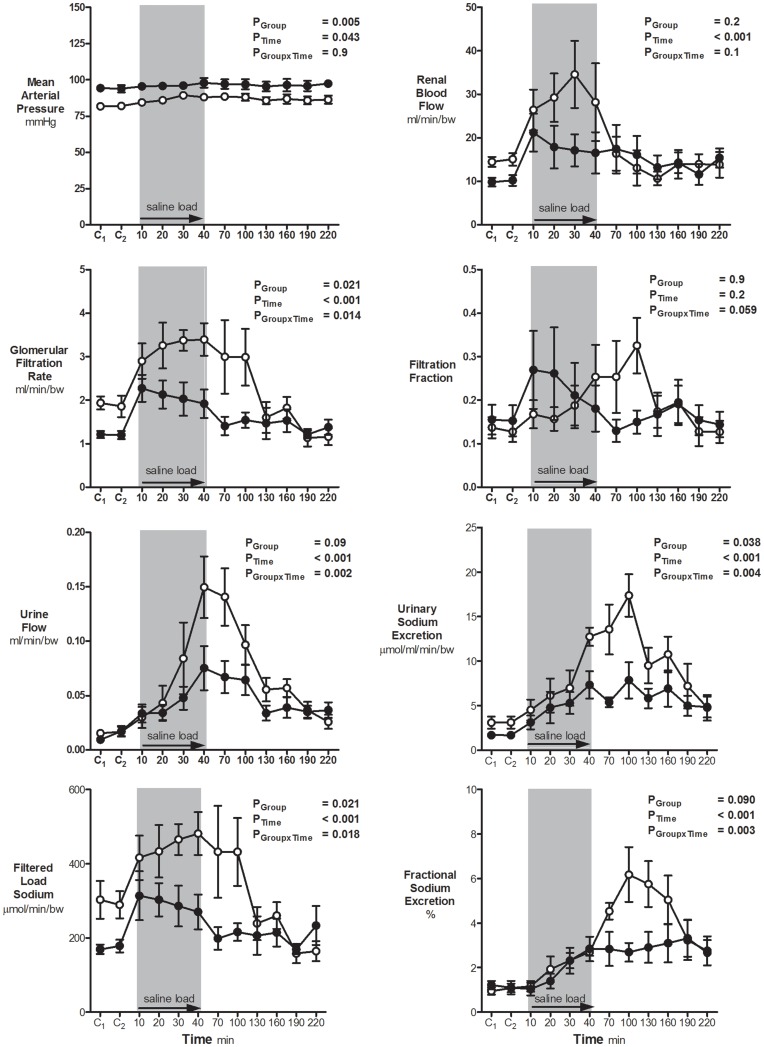
Cardiovascular and renal function responses to a saline load. Variables (mean ± S.E.M) were measured at hourly intervals over two control (basal) periods (C_1_ & C_2_), followed by intravenous saline loading (25 ml/kg/40 min) and a 3 hour recovery period in 5 year old sham (n = 7; open circles) and uni-x (n = 7; closed circles) female sheep. All renal variables are presented as absolute values corrected for body weight. P values represent the results of a repeated measures ANOVA, with factors group, (sham, uni-x), time and their interaction.

In response to the saline load there was a small but significant increase in MAP over time in both the uni-x and sham animals (P_Time = _0.04; Figure 2). The basal difference in MAP between groups was maintained throughout the study (P_Group_ = 0.005; Figure 2) and the effect of the saline load on MAP was not significantly different between the groups (P_GroupxTime_ = 0.9; Figure 2). MAP increased by the end of the 40 minute saline infusion by 5±3 mmHg in the sham group and 3±2 mmHg in the uni-x group. Heart rate was not significantly altered in response to the saline load in either group (data not shown).

#### Urine flow and sodium excretion

**Figure 3:**
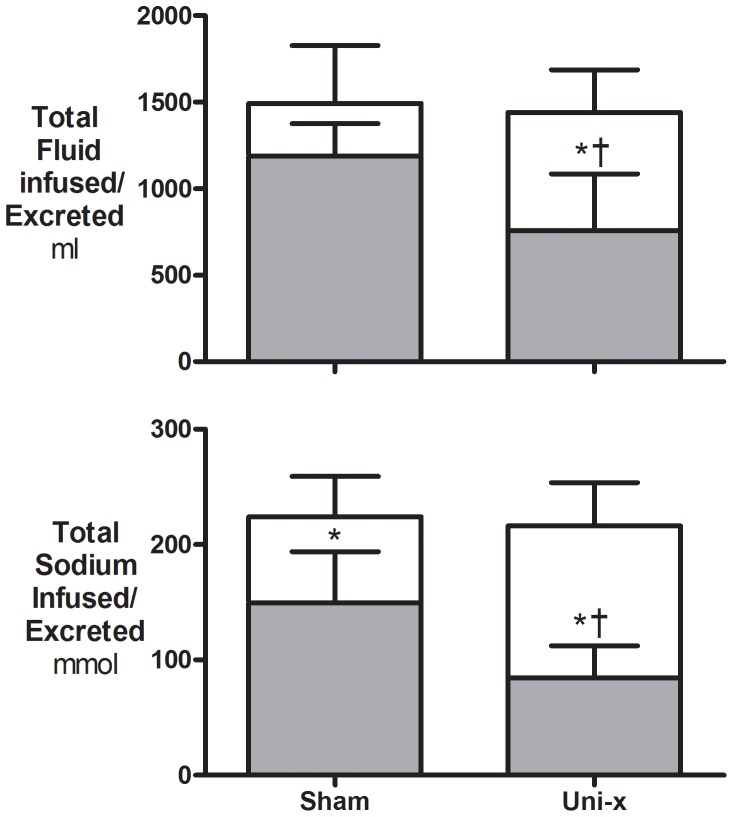
Total fluid volume and amount of sodium infused and excreted during the saline load and recovery period. Total fluid volume and the amount of sodium infused (open bar) and excreted (grey bar) during the 40-minutes of saline loading and the 3-hour recovery period in 5 year old sham (n = 7) and uni-x (n = 7) female sheep. Variables are expressed as mean ± S.E.M. * P<0.05 total volume/amount of sodium infused to that excreted within a group or † P<0.05 between groups from a two-tailed Student’s t test.

UF increased significantly in both groups in response to the saline load. However the increase in UF was markedly attenuated in the uni-x animals as compared to the sham animals (P_Group = _0.09, P_Time_<0.001, P_GroupxTime_ = 0.002; Figure 2). U_Na+_V increased in response to the saline load, and this response was noticeably reduced in the uni-x animals compared to the shams (P_Group = _0.04, P_Time_<0.001, P_GroupxTime_ = 0.004; Figure 2). As body weight was similar in the uni-x and sham animals, the volume of saline (0.9% NaCl) infused was equivalent in both groups (Figure 3). Neither the sham or uni-x sheep excreted the full saline load by the end of the 3-hour recovery period (Figure 3). The uni-x retained a significantly higher percentage of the total volume (sham, 22±9%; uni-x, 48±7%, P = 0.03; Figure 3) and sodium (sham, 32±6%; uni-x, 60±5%, P = 0.006; Figure 3) infused as compared to the sham animals.

#### Glomerular filtration rate, renal blood flow and filtration fraction

GFR (P_Time_<0.001) and RBF (P_Time_<0.001) increased in response to the acute saline load and returned to basal levels by the end of the recovery period (Figure 2). However, the increase in GFR (P_GroupxTime_ = 0.014) was significantly attenuated in the uni-x compared to the sham group. Overall, the RBF response to the saline load was not significantly different between the groups (P_GroupxTime_ = 0.1). However, post-hoc analysis demonstrated that the absolute increase in RBF at 30 minutes into the saline load was significantly less in the uni-x sheep as compared to the shams (P<0.05). There was no significant change in FF in response to the saline load in sham and uni-x sheep (P_Group = _0.9, P_Time = _0.2, P_GroupxTime_ = 0.06; Figure 2).

#### Filtered load and fractional excretion of sodium

The filtered load of sodium increased significantly in both the sham and uni-x animals in response to the saline load, though the increase was attenuated in the uni-x sheep (P_Group = _0.02, P_Time_<0.001, P_GroupxTime_ = 0.018, Figure 2). This increase in filtered sodium load was due to the increase in GFR, since plasma sodium concentration was not significantly different between the groups (data not shown). Both sham and uni-x animals increased their FE_Na+_ in response to the saline infusion (Figure 2), but this increase was markedly attenuated in the uni-x animals (P_Group = _0.09, P_Time_<0.001, P_GroupxTime_ = 0.003; Figure 2).

#### Plasma renin activity

**Figure 4:**
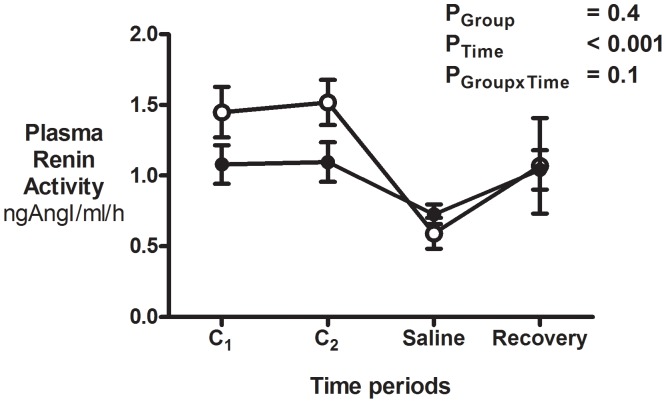
Plasma renin activity in response to an acute saline load. PRA was measured at hourly intervals at the end of the two control (basal) periods (C_1_ & C_2_), at the end of saline load at 40 minutes and at the end of the 3-hour recovery period in 5 year old sham (n = 7; open circles) and uni-x (n = 7; closed circles) female sheep. Variables are expressed as mean ± S.E.M. P values represent the results of a repeated measures ANOVA, with factors group, (sham, uni-x), time and their interaction.

Basal PRA was ∼27% lower in the uni-x as compared to the sham sheep (P = 0.013). PRA dropped to the same absolute level in the sham and uni-x groups by the end of the infusion of the saline load at 40 minutes (P_Time_<0.001; Figure 4). Post-hoc analysis demonstrated that since basal PRA was lower in the uni-x sheep, the fall in PRA was 23±13% in the uni-x as opposed to a 58±8% reduction in the sham sheep (P = 0.042) in response to the saline load at 40 minutes. At the end of the 3-hour recovery period, PRA had increased by 22±10% from the nadir at the end of the saline load in the uni-x and by 34±9% in the sham animals (P>0.05, Figure 4).

#### Renal gene expression

**Figure 5:**
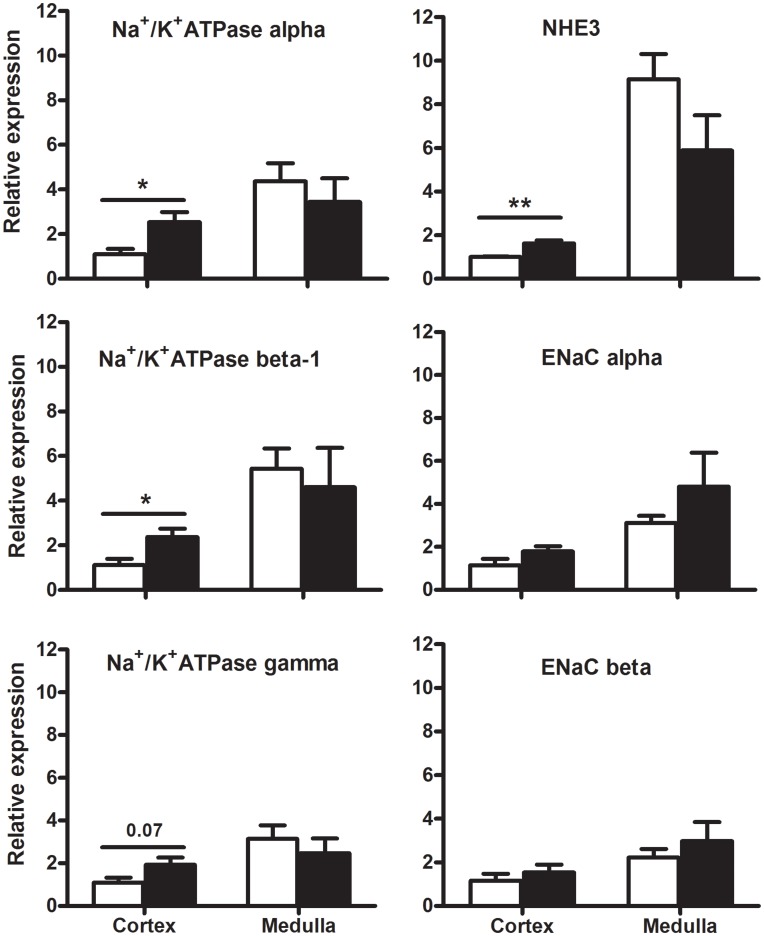
Effect of fetal uni-x on renal cortical and medullary mRNA expression of Na^+^/K^+^ ATPase subunits α, β and γ, NHE3 and the ENaC subunits α and β in female sheep at 5 years of age. Gene expression is presented relative to the housekeeping gene 18S in sham (n = 5; open bars) and uni-x (n = 5; closed bars) sheep. Values are expressed as mean ± S.E.M. P values represent results from a two-tailed Student’s t test. * P<0.05 and ** P<0.01.

The uni-x animals had a significantly higher expression of both the Na^+^/K^+^ ATPase α and β1 subunits in their renal cortex compared to the sham animals (P<0.05; Figure 5). However the expression of Na^+^/K^+^ ATPase γ subunit in the renal cortex was not significantly different (P = 0.07; Figure 5), nor was the expression of the Na^+^/K^+^ ATPase subunits (α, β and γ) in the renal medulla significantly different between the two treatment groups. The expression of NHE3 was significantly greater in the renal cortex (P = 0.003), but not the renal medulla of the uni-x as compared to the sham sheep (Figure 5). The expression of the ENaC (α and β) subunits in the renal cortex and medulla, were not significantly different between the uni-x and sham animals (Figure 5).

## Discussion

The major finding of this study was that 5-year old female sheep that had undergone uni-nephrectomy at 100 days of gestation had a significantly blunted ability to excrete a saline load. It was determined that the reduced capacity to excrete sodium in the uni-x as compared to the sham sheep was due to both a reduced absolute increase in the filtered load of sodium and fractional excretion of sodium. Moreover, it was observed that neither the uni-x nor the sham 5-year old sheep could completely excrete the saline load within the time course of the study. In addition, basal PRA was lower in the uni-x sheep, and thus whilst PRA decreased in both sham and uni-x animals in response to the saline load to a similar nadir, the fall was less in the uni-x sheep. This reduced basal PRA in the uni-x sheep may represent an appropriate homeostatic response; that is suppression of the systemic RAS to counter the increase in arterial pressure. It is also possible that the reduction in PRA may be directly related to the reduced nephron number, which would inherently be associated with a reduction in the number of juxtaglomerular apparatus present within the uni-x kidney and perhaps a reduced ability to produce renin. It would appear that the reduced gain of the RAS may compromise the ability of offspring with a congenital nephron deficit to mount a response to an extracellular fluid volume expansion.

The volumes of saline delivered to sham and uni-x groups were not significantly different. However, the cumulative volume of fluid (52% vs 78%) and the amount of sodium (40% vs 68%) excreted over the 3 hours following 40 minutes of saline loading was reduced in the uni-x in comparison to the sham sheep respectively. Notably, neither group was able to excrete the complete saline load within the time frame of the study, suggesting an age-related decline in the ability to respond to an expansion of the extracellular fluid volume. Previously, we demonstrated that in 6 month old males, both the sham and uni-x sheep were able to excrete a larger saline load (5% bodyweight) completely within a 3 hour recovery period, albeit more slowly in the uni-x [13]. These observations suggest that younger sheep are capable of excreting a volume load and correcting extracellular fluid homeostasis more efficiently than aged sheep. There is evidence demonstrating an inverse relationship between age and GFR [21], [23], [30]–[34]. This age-dependent decline in GFR has been shown to be a consequence of decreases in renal plasma flow, the glomerular capillary ultrafiltration coefficient (K_f_) and reductions in the number of functional glomeruli [32], [34]. Furthermore, loss of glomeruli with age has been shown to occur continuously throughout adult life, with a mean predicted loss of ∼4500 glomeruli per kidney per year occurring between ages 18 and 70 years in humans [24]. The lifespan of a sheep averages 10–12 years, thus the sheep in the current study could be considered middle aged. Although we have previously demonstrated no age-related decline in GFR in female sheep between 1–5 years of age [19], our present study suggests that at 5 years of age the functional capacity of the kidney to respond to a physiological challenge has declined in both the sham and uni-x animals.

This impairment in the regulation of volume homeostasis was exacerbated in animals born with a congenital nephron deficit. The renal response to the saline load was markedly blunted, with the uni-x sheep retaining 26% more of the saline volume than the sham sheep. The excretion of a saline load depends on the kidney’s ability to increase GFR (i.e. increase the filtered load of sodium) and/or decrease the net tubular reabsorption of sodium, in order to regulate sodium balance and maintain extracellular fluid homeostasis [35], [36]. Our study demonstrated that increases in GFR and decreases in the tubular sodium reabsorption contributed to the natriuresis observed in both the sham and uni-x sheep in response to the saline load. However, both of these responses were attenuated in the uni-x sheep. The absolute increase in GFR and RBF were significantly reduced in the uni-x sheep in response to the saline load as compared to the sham group. The uni-x sheep lost 50% of their renal mass at 100 days of gestation, however, at completion of nephrogenesis it was previously demonstrated that there is degree of compensation following uni-nephrectomy with the total nephron deficit being only ∼30% at 130 days of gestation (time when nephrogenesis is complete in sheep) [10]. Therefore, it appears that the degree to which GFR increased in response to the saline load while reduced in absolute terms in the uni-x sheep was proportionate to nephron number, with both groups increasing GFR by ∼70% above baseline. This suggests that the functional reserve of the individual nephrons has been preserved in the uni-x sheep at 5 years of age. These observations agree with studies carried out in healthy elderly humans (up to 80 years of age) [37] and in long term survivors of unilateral Wilms tumour (15 years post-nephrectomy) [38]; revealing that despite reductions in both GFR and RBF, renal functional reserve can still be preserved with ageing. Nevertheless, the reduced nephron number and the associated absolute reduction in the increase in GFR in response to a saline load, has had an impact on how rapidly extracellular fluid homeostasis can be maintained in the uni-x sheep.

In response to the saline load, urinary sodium excretion was lower in uni-x sheep compared to the sham and this was due in part to an attenuated rise in fractional sodium excretion in the uni-x animals. We have previously reported that the remnant kidney from these female uni-x sheep was significantly larger than a single kidney from the sham group [19]. Therefore, it is likely that significant compensatory tubular growth may, in part, explain the increases in tubular sodium reabsorption seen in the uni-x animals. Nephrectomy in rats has been shown to result in the increase of proximal and distal tubular lengths by approximately 35% and 17%, which may result in augmented sodium reabsorption [39], [40]. The present study demonstrates greater gene expression of the apical NHE3 and basolateral Na^+^/K^+^ ATPase mRNA (α and β subunits) transporters in the renal cortex of uni-x female sheep at 5 years of age, which is compatible with our findings in 6 month old uni-x male sheep, where renal mRNA expression of Na^+^/K^+^ ATPase subunits, and the apical NHE3 and ENaC β and γ subunits were all up-regulated [13]. Hence, an up-regulation in expression of tubular sodium transporters may have contributed to the blunted ability to excrete the saline load in the uni-x sheep. Mounting evidence demonstrates a role for increased sodium transport activity in models of programmed hypertension, for example up-regulation of sodium transporters have been reported in 2 month old female sheep born with a ∼30% congenital nephron deficit following a prenatal exposure to glucocorticoids, contributing to their development of hypertension in adulthood [4]. A limitation of these findings is that mRNA gene expression does not necessarily reflect an increase in gene transcription. Furthermore, even if transcribed, these transporters may not be located in the cell membrane, which would be necessary for increased functional activity. Therefore, further studies are required to elucidate the functional impact of this increase in sodium transporters in the uni-x kidney. However, previously, in young adult rats following uninephrectomy NHE3 gene expression data was demonstrated to correlate with alterations in NHE3 protein levels and activity [41]. Although, the hypertension observed in these uni-x sheep may result directly from a reduced filtered load, as a result of a reduced nephron number and GFR, along with a reduction in urinary sodium excretion, the mechanisms are likely to be more complex and involve other intrarenal mechanisms.

The RAS is a powerful modulator of body fluid homeostasis and long-term blood pressure [42], [43]. Age-dependent alterations in the renal RAS have been documented in several models of fetal programming [1], [5], [14]. Overall, these studies indicate a decrease in the activity of the RAS in models of impaired nephrogenesis [1], [14]. In the uni-x male sheep blood volume is increased compared to the shams [11] and this is associated with a lower basal PRA [12], [13]. In the present study, since basal PRA was reduced by ∼27%, the magnitude of the normal down-regulation of the RAS in response to a saline load was reduced in the uni-x sheep compared to the shams. These results are consistent with studies in low renin essential hypertensive patients in which delays in diuresis and natriuresis are observed in response to volume loading [44]–[47]. Thus, the low basal PRA in our model suggests that the RAS is appropriately down-regulated with regard to the elevation in arterial pressure; however, it is possible that this down-regulation has contributed to the reduced ability of the uni-x to excrete a saline load. Alternatively, it is possible that despite systemic down-regulation, intrarenal RAS may be enhanced as demonstrated in other fetal programming models of hypertension [48]. In addition, we have previously reported, that there were no gross morphological changes in the renal architecture in uni-x sheep, which might have contributed to the low PRA [49]. However, these possibilities warrants further investigation, given that in response to angiotensin receptor blockade in young male uni-x sheep we observed a marked increase in the fractional sodium excretion [12].

Finally, pressure-natriuresis plays a dominant role in the long-term control of arterial pressure and body fluid balance [50]–[52]. The present study demonstrates that in response to the saline load there was a modest rise in arterial pressure over time in the sham (∼5 mmHg) and uni-x (∼3 mmHg) sheep. Similar elevations in arterial pressure (2–6 mmHg) have been reported in conscious normotensive dogs in response to isotonic saline loading [53], [54]. Under normal conditions pressure-natriuresis acts to normalize arterial pressure, however, abnormalities in renal hemodynamics or tubular reabsorption have been shown to shift the pressure natriuresis curve to higher pressure settings [52]. In the present study we showed that despite similar rises in arterial pressure during saline loading in both groups, the uni-x sheep exhibited significant reductions in fluid and sodium excretion compared to the shams, suggesting a rightward shift in the pressure-natriuresis relationship. This result is consistent with other models of hypertension [4], [52], [55], where studies carried out in rats exposed to a low-protein diet prenatally, have revealed rightward shifts in the pressure-natriuresis curves when their blood pressure was ramped across the autoregulatory range [55]. Similar findings have also been reported in 4–5 year old sheep models of congenital nephron deficit [4]. Thus, future studies need to be focused on examining some of the underlying renal intrinsic mechanisms responsible for the impaired renal function in sheep born with a low nephron endowment.

### Conclusion

There is currently very limited literature on the long-term repercussions of being born with a low nephron endowment on the progression of cardiovascular disease in more clinically relevant, aged cohorts. A congenital nephron deficit brought about by fetal uninephrectomy (uni-x) leads to the development of hypertension and a reduction in renal function by 2 years of age in intact female sheep [19]. Furthermore, we have recently provided evidence suggesting that these changes do not progress with age [19]. However, the current study demonstrates perturbations in the way in which the uni-x sheep regulate renal function in response to changes in extracellular fluid volume, revealing not only reductions in GFR of the solitary uni-x kidney, but also alterations in tubular sodium reabsorption as well as a reduced contribution of the RAS to changes in body fluid volumes. These impairments in the ability of the kidney to regulate extracellular fluid homeostasis may render adults with a congential nephron deficit increasingly vulnerable to secondary renal insults in postnatal life, such as obesity, diabetes and alterations in dietary salt intake.
